# Elevated Dehydroepiandrosterone Sulfate Levels in 2 Patients With Prolactinomas: An Underrecognized Association

**DOI:** 10.1210/jcemcr/luaf111

**Published:** 2025-05-15

**Authors:** Joseph Arguinchona, Aditi Kumar, Divya Yogi-Morren, Mona Vahidi Rad, Krupa Doshi

**Affiliations:** Department of Endocrinology, Mayo Clinic Arizona, Scottsdale, AZ 85259, USA; Department of Endocrinology, Mayo Clinic Arizona, Scottsdale, AZ 85259, USA; Department of Endocrinology, Cleveland Clinic, Cleveland, OH 44195, USA; Department of Endocrinology, Mayo Clinic Arizona, Scottsdale, AZ 85259, USA; Department of Endocrinology, Mayo Clinic Arizona, Scottsdale, AZ 85259, USA

**Keywords:** DHEA-S, prolactinoma, hyperprolactinemia, adrenal androgens

## Abstract

While adrenal androgen production is primarily regulated by ACTH, some data suggest a less common association between hyperprolactinemia and elevated dehydroepiandrosterone sulfate (DHEA-S). We describe 2 patients with this underrecognized connection. Patient 1, a 60-year-old male, was incidentally found to have bilateral adrenal masses on computed tomography during evaluation of gastrointestinal complaints. Further workup revealed elevated levels of DHEA-S and prolactin, with pituitary macroadenoma identified as the cause, given normalization of both prolactin and DHEA-S on cabergoline therapy. Patient 2, a 38-year-old male, presented with visual field defects, and subsequent pituitary magnetic resonance imaging confirmed a macroadenoma. He also had markedly elevated levels of DHEA-S and prolactin. Both patients were treated with cabergoline, leading to rapid normalization of both prolactin and DHEA-S levels. These cases demonstrate an association between hyperprolactinemia and elevated DHEA-S, supported by the normalization of levels with medical treatment of prolactinoma. Although hyperprolactinemia in the context of a prolactinoma is an uncommon cause of elevated DHEA-S, it should be considered once other etiologies have been excluded.

## Introduction

Dehydroepiandrosterone sulfate (DHEA-S) is produced in the zona reticularis, the innermost layer of the adrenal cortex, and its production is primarily regulated by ACTH [1]. However, hyperprolactinemia has been associated with elevated DHEA-S, suggesting that prolactin may also play a role in adrenal androgen secretion. Previous literature has demonstrated this phenomenon through reversible and reproducible changes in DHEA-S levels following medical and surgical treatment of prolactinomas [2].

The exact mechanism by which this occurs is not fully elucidated [2, 3]. Several proposed mechanisms include increased sulfokinase activity, decreased 17,20-desmolase activity, and decreased 3-β-hydroxysteroid dehydrogenase activity, all of which may contribute to elevated adrenal androgens [2, 4, 5]

While multiple reports have documented a reduction in DHEA-S with therapeutic lowering of prolactin, several older studies have shown no effect of prolactin reduction on adrenal androgen secretion [6, 7]. Many of these earlier studies were limited by small sample sizes and included patients with varying causes of hyperprolactinemia.

We describe 2 male patients, aged 60 and 38, both diagnosed with macroprolactinomas accompanied by elevated DHEA-S levels. Treatment with cabergoline successfully normalized prolactin and DHEA-S levels in both patients.

## Case Presentation

### Patient 1

A 60-year-old male with a history of Crohn’s disease underwent abdominal computed tomography as part of a gastrointestinal workup, which incidentally showed bilateral adrenal masses measuring 4.6 × 3.9 cm on the left (unenhanced attenuation value 25 Hounsfield units) and 1.2 × 2.7 cm on the right (unenhanced attenuation value 19 Hounsfield units). He was referred to endocrinology for further evaluation. He denied headaches, palpitations, flushing, pallor, weight loss, or hypertension, though he reported midday fatigue. A physical exam was unremarkable.

### Patient 2

A 38-year-old male with a 10-year history of hypogonadism on testosterone replacement was referred to endocrinology after developing bilateral visual field defects. Brain magnetic resonance imaging (MRI) at an outside facility showed a 3.1 × 2.0 × 2.7 cm macroadenoma.

## Diagnostic Assessment

### Patient 1

Initial hormonal evaluation revealed a normal renin-aldosterone axis, normal plasma metanephrine, and an appropriately suppressed cortisol after an overnight low-dose dexamethasone suppression test. DHEA-S levels were markedly elevated at 751.3 μg/dL [20.4 μmol/L] (reference 10.0-204.0 μg/dL [0.3-5.5 μmol/L]). To rule out nonclassical congenital adrenal hyperplasia in the setting of bilateral adrenal masses and elevated DHEA-S, an ACTH stimulation test was performed; it was within normal limits. His total testosterone was low at 142 ng/dL [4.9 nmol/L] (reference 220-1000 ng/dL [7.6-34.7 nmol/L]) while free testosterone was normal at 41.7 ng/dL [1.45 nmol/L] (reference 40-240 ng/dL [1.39-8.32 nmol/L]). Given low testosterone levels, a prolactin level was checked and noted to be significantly elevated at 683.6 ng/mL [29.7 nmol/L] (reference 2.0-14.0 ng/mL [0.09-0.61 nmol/L]) (Table 1).

**Table 1.** Initial laboratory studies for patient 1

Laboratory studies	Reference ranges	Results
Aldosterone	4.5-35.4 ng/dL [124.8-982.0 pmol/L]	7.7 ng/dL [213.6 pmol/L]
Plasma renin activity	Upright = 0.8-5.8 μg/L/hr, Supine = 0.5-1.8 μg/L/hr [Upright = 0.8-5.8 ng/mL/hr, Supine 0.5-1.8 ng/mL/hr]	0.9 μg/L/hr [0.9 ng/mL/hr]
Plasma metanephrine	12-67 pg/mL [60.8-339.7 pmol/L]	46 pg/mL [233.2 pmol/L]
Cortisol*^a^*	< 1.8 μg/dL [<49.7 nmol/L]	< 1.8 μg/dL [<49.7 nmol/L]
DHEA-S	10.0-204.0 μg/dL [0.3-5.5 μmol/L]	751.3 μg/dL [20.4 μmol/L]
17-OHP baseline*^b^*	0.4-1.8 ng/mL [0.01-0.05 nmol/L]	1.3 ng/mL [0.04 nmol/L]
17-OHP, 60 minutes post*^b^*	2.0-14.0 ng/mL [0.06-0.42 nmol/L]	5.2 ng/mL [0.16 nmol/L]
Total testosterone	220-1000 ng/dL [7.6-34.7 nmol/L]	142 ng/dL [4.9 nmol/L]
Free testosterone	40-240 ng/dL [1.39-8.32 nmol/L]	41.7 ng/dL [1.45 nmol/L]
Prolactin	2.0-14.0 ng/mL [0.09-0.61 nmol/L]	683.6 ng/mL [29.7 nmol/L]

Abbreviations: 17-OHP, 17-hydroxyprogesterone; DHEA-S, dehydroepiandrosterone sulfate.

^*a*^Cortisol drawn after 1-mg overnight dexamethasone suppression test.

^*b*^17-OHP levels are listed at baseline and 60 minutes after administration of ACTH.

**Figure 1.**
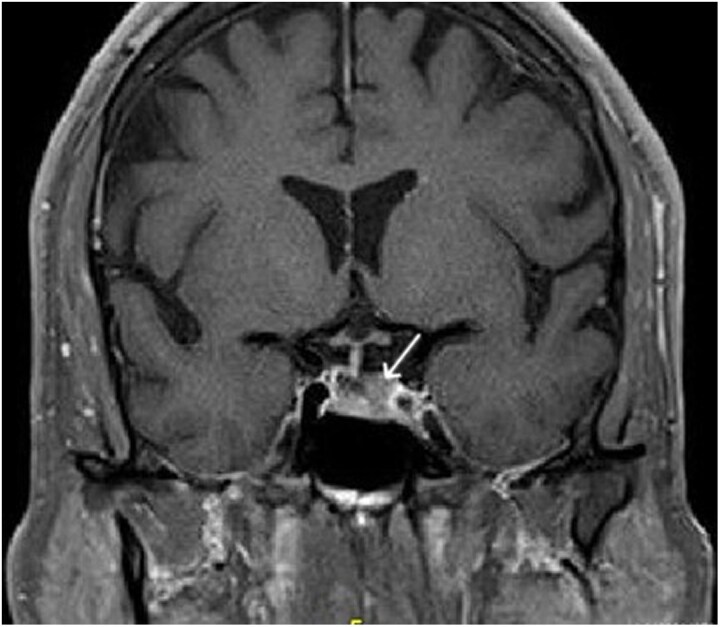
Pituitary magnetic resonance imaging before treatment with cabergoline for patient 1. Here, the left-sided pituitary macroadenoma is visualized, resulting in deviation of the gland and stalk to the right.

A pituitary MRI showed a 0.9 × 1.5 × 0.6-cm left-sided pituitary macroadenoma causing deviation of the gland and stalk to the right, abutting but not encasing the left cavernous sinus. There was no mass effect on the optic chiasm (Fig. 1). Regarding his bilateral adrenal nodules, through shared decision-making between the patient and his provider, it was decided to pursue active imaging surveillance rather than biopsy.

### Patient 2

**Figure 2.**
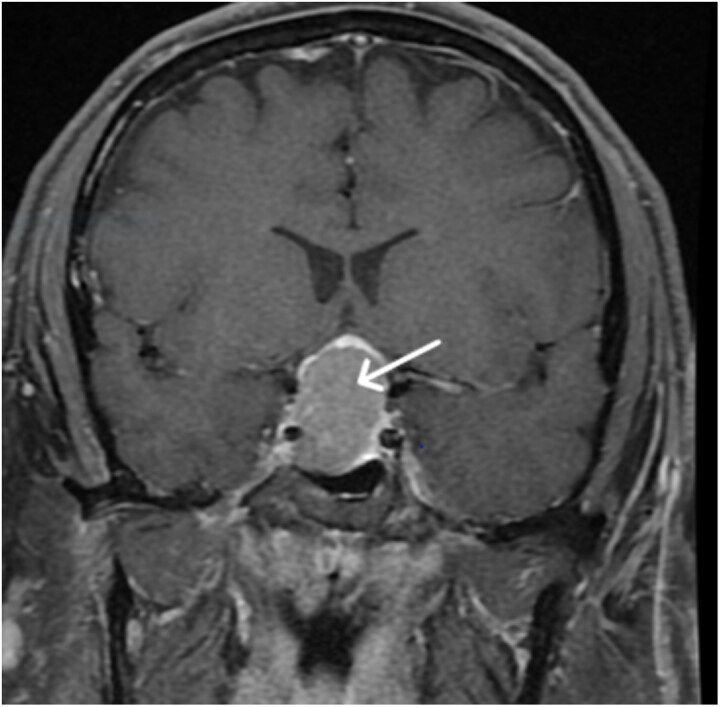
Magnetic resonance imaging of the brain before treatment with cabergoline for patient 2. Here, the pituitary macroadenoma is visualized bulging into the right cavernous sinus and resulting in mass effect on the optic chiasm.

This patient had a known macroadenoma, measured at 3.1 × 2.0 × 2.7 cm and associated with mass effect on the optic chiasm, abutting the cavernous right internal carotid artery (Fig. 2). His initial hormonal evaluation revealed a free testosterone of 20.5 ng/dL [0.71 nmol/L] (reference 40-240 ng/dL [1.39-8.32 nmol/L]), total testosterone of 437 ng/dL [15.16 nmol/L] (reference 220-1000 ng/dL [7.6-34.7 nmol/L]) (measured midcycle on testosterone 80 mg weekly), and prolactin of 3314 ng/mL [144 nmol/L] (reference 2.0-14.0 ng/mL [0.09-0.61 nmol/L]). Multiple DHEA-S levels ranged from 744 to 912 μg/dL [20.2-24.7 μmol/L] (reference 57-522 μg/dL [1.5-14.1 μmol/L]) (Table 1). Cortisol, ACTH, IGF-1, free T4, and TSH were within normal limits. LH and FSH were suppressed in the setting of exogenous testosterone use (Table 2). Unenhanced computed tomography of the abdomen showed normal-appearing adrenal glands.

**Table 2.** Initial laboratory studies for patient 2

Laboratory studies	Reference ranges	Results
IGF-1	48-292 ng/mL [6.3-38.3 nmol/L]	204 ng/mL [26.7 nmol/L]
ACTH	7.2-63 pg/mL [1.6-13.9 pmol/L]	57 pg/mL [12.5 pmol/L]
Morning cortisol	4.8-20 μg/dL, [132.4-551.7 nmol/L]	9.4 μg/dL [259.3 nmol/L]
DHEA-S	57-522 μg/dL [1.5-14.1 μmol/L]	912 μg/dL [24.7 μmol/L]
FSH	1.2-15.8 IU/L	<0.3 IU/L
LH	1.3-9.6 IU/L	<0.2 IU/L
Total testosterone	220-1000 ng/dL [7.6-34.7 nmol/L]	437 ng/dL [15.16 nmol/L]
Free testosterone	40-240 ng/dL [1.39-8.32 nmol/L]	20.5 ng/dL [0.71 nmol/L]
Prolactin	2.0-14.0 ng/mL [0.09-0.61 nmol/L]	3314 ng/mL [144 nmol/L]
TSH	0.3-4.2 mIU/L	1.2 mIU/L
Free thyroxine	0.9-1.7 ng/dL [11.6-21.9 pmol/L]	1.2 ng/dL [15.4 pmol/L]

Abbreviation: DHEA-S, dehydroepiandrosterone sulfate.

## Treatment

### Patient 1

Cabergoline was initiated at 0.25 mg twice per week for treatment of his prolactinoma. The dose was subsequently increased to 0.5 mg twice per week.

### Patient 2

Cabergoline was started at 0.5 mg twice per week for prolactinoma treatment. In addition, the patient was advised to discontinue his testosterone injections, to which he agreed.

## Outcome and Follow-up

### Patient 1

**Figure 3.**
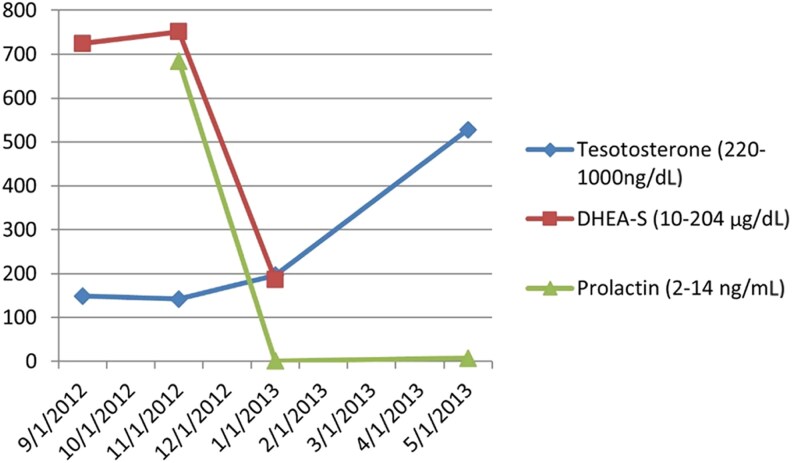
Prolactin, dehydroepiandrosterone sulfate, and testosterone levels before and after treatment with cabergoline for patient 1.

The patient had an excellent response to cabergoline over 2 months (Fig. 3). His prolactin level decreased from 683.6 ng/mL [29.7 nmol/L] to 0.9 ng/mL [0.04 nmol/L] (reference 2.0-14.0 ng/mL [0.09-0.61 nmol/L]). Serum DHEA-S level decreased from 751.3 μg/dL [20.4 μmol/L] to 186.6 μg/dL [5.1 μmol/L] (reference 10.0-204.0 μg/dL [0.3-5.5 μmol/L]). Total testosterone increased from 142 ng/dL [4.9 nmol/L] to 528 ng/dL [18.3 nmol/L] (reference 220-1000 ng/dL [7.6-34.7 nmol/L]). Adrenal nodules remained stable on follow-up imaging.

### Patient 2

**Figure 4.**
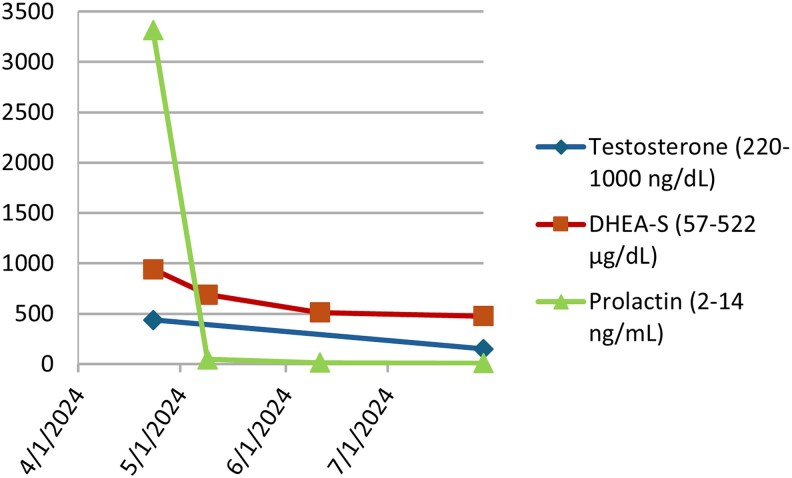
Prolactin, dehydroepiandrosterone sulfate, and testosterone levels before and after treatment with cabergoline for patient 2. Notably, he was on testosterone replacement prior to the initial visit, which was stopped.

The patient had an excellent response to cabergoline therapy over several months (Fig. 4). His prolactin level deceased to 14.5 ng/mL [0.63 nmol/L] (reference 2.0-14.0 ng/mL [0.09-0.61 nmol/L]). DHEA-S levels also normalized to 477 μg/dL [13.9 μmol/L] (reference 57-522 μg/dL [1.5-14.1 μmol/L]). Total testosterone levels were low at 151 ng/dL [5.24 nmol/L] (reference 220-1000 ng/dL [7.6-34.7 nmol/L]), with a free testosterone of 5.18 ng/dL [0.18 nmol/L] (reference 40-240 ng/dL [1.39-8.32 nmol/L]), as expected, due to prior long-term exogenous testosterone replacement.

Pituitary MRI 1 month after beginning therapy showed the macroprolactinoma decreased in size from 3.1 × 2.0 × 2.7 cm to 1.9 × 1.6 × 2.7 cm. Visual field testing revealed almost complete resolution of bilateral hemianopsia within 2 weeks of starting cabergoline.

## Discussion

In both cases, patients exhibited a reversible association between hyperprolactinemia, secondary to prolactinomas, and elevated DHEA-S levels. While hyperprolactinemia is a recognized reversible cause of secondary hypogonadism, it is less often considered a cause of increased DHEA-S. Other differential considerations include nonclassical congenital adrenal hyperplasia, adrenal adenomas or carcinomas, and Cushing syndrome in the presence of suggestive clinical features [8].

Recent literature supports the association between hyperprolactinemia and increased DHEA-S. A 2019 study by Moria et al examined 148 patients with prolactinoma [2]. Of these, 122 were treated medically and 26 underwent surgery. Serum prolactin and DHEA-S levels were measured before and after treatment at consistent time intervals. The study found that for medically treated patients with normal hypothalamic-adrenal-pituitary function, serum prolactin levels significantly decreased from 728 ± 1507 μg/L to 29.1 ± 39 μg/L (*P* < .001) at 3 months, which was accompanied by a significant reduction in DHEA-S levels from 245.9 ± 196 μg/dL to 216.2 ± 203.3 μg/dL (*P* < .001) at 3 months. Similar changes in DHEA-S levels were observed in surgically treated patients, indicating that the reduction in DHEA-S was attributable to the decrease in prolactin rather than the effect of dopamine agonists [2].

A 2001 study by Hagag et al assessed 80 women with hyperprolactinemia who presented with hirsutism. After 11 ± 1 months of bromocriptine therapy, prolactin and DHEA-S levels decreased by 40% to 85% during dopaminergic treatment (*P* < .001) [3].

Although most studies support an association between hyperprolactinemia and elevated DHEA-S, some studies do not corroborate this finding [6, 7]. However, many of these studies are limited by the diversity of causes of hyperprolactinemia and small patient samples [2].

These cases highlight hyperprolactinemia as a potential cause of elevated adrenal androgens, demonstrated by normalization of DHEA-S following cabergoline treatment for both patients’ prolactinomas. The key takeaway of these cases is that hyperprolactinemia should be considered in the differential diagnosis of elevated DHEA-S, particularly when more common causes such as adrenal tumors and congenital adrenal hyperplasia have been ruled out.

## Learning Points

- Hyperprolactinemia can be associated with elevated levels of DHEA-S.
- Prolactinoma should be considered in the differential of elevated DHEA-S, particularly when other more common causes are ruled out.
- Effective treatment of prolactinoma, often with medications that reduce prolactin levels, can result in normalization of DHEA-S levels.

## Contributors

All authors made individual contributions to authorship. K.D. and D.Y. were involved in the diagnosis and management of this patient as well as manuscript submission. A.K., K.D., D.Y., J.A., and M.V. were involved with the draft and review of the manuscript for submission. All authors reviewed and approved the final draft.

## Data Availability

Data sharing is not applicable to this article as no datasets were generated or analyzed during the current study.
